# Occludin facilitates tumour angiogenesis in bladder cancer by regulating IL8/STAT3 through STAT4

**DOI:** 10.1111/jcmm.17257

**Published:** 2022-02-27

**Authors:** Fan Yang, Xue‐Qi Liu, Jian‐Zhong He, Shi‐Ping Xian, Peng‐Fei Yang, Zhi‐Ying Mai, Miao Li, Ye Liu, Xing‐Ding Zhang

**Affiliations:** ^1^ Department of Pathology The Fifth Affiliated Hospital of Sun Yat‐Sen University Zhuhai China; ^2^ Molecular Cancer Research Center, School of Medicine, Shenzhen Campus of Sun Yat‐Sen University Sun Yat‐Sen University Shenzhen China; ^3^ Department of Hematology The First Affiliated Hospital of Shenzhen University, Shenzhen Second People’s Hospital Shenzhen China

**Keywords:** bladder cancer, IL8, OCLN, tumour angiogenesis

## Abstract

Bladder cancer (BLCA) is a common genitourinary cancer in patients, and tumour angiogenesis is indispensable for its occurrence and development. However, the indepth mechanism of tumour angiogenesis in BLCA remains elusive. According to recent studies, the tight junction protein family member occludin (OCLN) is expressed at high levels in BLCA tissues and correlates with a poor prognosis. Downregulation of OCLN inhibits tumour angiogenesis in BLCA cells and murine xenografts, whereas OCLN overexpression exerts the opposite effect. Mechanistically, the RT‐qPCR analysis and Western blotting results showed that OCLN increased interleukin‐8 (IL8) and p‐signal transducer and activator of transcription 3 (STAT3) levels to promote BLCA angiogenesis. RNA sequencing analysis and dual‐luciferase reporter assays indicated that OCLN regulated IL8 transcriptional activity via the transcription factor STAT4. In summary, our results provide new perspectives on OCLN, as this protein participates in the development of BLCA angiogenesis by activating the IL8/STAT3 pathway via STAT4 and may serve as a novel and unique therapeutic target.

## 1 INTRODUCTION

Bladder cancer (BLCA) is the most common malignant cancer of the urinary system. The incidence rate of BLCA in males is 3–4 times that in women, and it is the fourth most common malignancy in men.^1^ Non‐muscle‐invasive BLCA (NMIBC) accounts for approximately 75% of all BLCA cases, while other cases are considered muscle‐invasive bladder cancer (MIBC). The 5‐year survival rate of patients with NMIBC is more than 85%, but upon relapse, easy progression to MIBC, which invades the basement membrane and spreads from the bladder to visceral organs, is likely and eventually leads to tumour metastasis and a low survival rate.^2^ Neovascularization contributes to growth and development, tissue and organ regeneration and numerous pathological conditions of tumors.^3^ In addition, neovascularization is indispensable for the development of malignant tumors^4^ and provides opportunities for distant metastasis.^5^ Tumour vascularization involves a variety of biological processes that are regulated by a wide range of secreted factors and signalling pathways. Among them, antibodies and tyrosine kinase inhibitors play an important role in antiangiogenic treatment for several types of cancer.^6^ BLCA is a highly vascularized disease, and although angiogenesis has been reported to participate in the development of BLCA,^7^, ^8^ its molecular mechanism and related signalling pathways are generally still unclear. Therefore, the biological function and specific mechanism of tumour angiogenesis in BLCA remain to be fully elaborated.

Occludin (OCLN) is an important tight junction protein that regulates cytoskeletal remodeling,^9^ it is reported to be aberrantly expressed in many malignant cancers during tumour progression and to contribute to apoptosis and metastasis. In lung cancer, downregulation of OCLN inhibits activation of the AKT/PI3K signalling pathway and cell proliferation, thus increasing tumour apoptosis in vitro and in vivo.^10^ In addition, OCLN levels in the peripheral blood have been used as a tumorigenic biomarker of cerebral oedema caused by blood‐brain barrier (BBB) damage in patients with brain tumors.^11^ Other tight junction proteins, such as tight junction protein 1 (TJP1) and Claudin‐5, have been indicated to regulate vascular abnormalities during tumour progression.^12^, ^13^, ^14^ A few studies have also shown roles of OCLN in modulating tumour angiogenesis and tube‐forming activity for tumour invasion.^15^, ^16^ OCLN S490 phosphorylation mediates VEGF to induce retinal endothelial cell proliferation and neovascularization.^17^ Exosomal miR‐25‐3p promotes vascular permeability and angiogenesis by regulating OCLN expression in endothelial cells.^18^ However, researchers have not clearly determined whether and how OCLN regulates BLCA angiogenesis.

Signal transducers and activators of transcription signalling (STATs) have been proven to be molecules that connect cytokine signalling with the regulation of important cellular mechanisms such as tumour cell survival and infiltration, inflammation and immunity.^19^ Recently, one of the STAT proteins, STAT3, was reported to play critical roles in BLCA cell viability and invasiveness.^20^, ^21^ Furthermore, miR‐153 mediates BLCA vascular remodelling and tryptophan metabolism by targeting IL6/STAT3/VEGF signaling.^22^ Another STAT, STAT4, has also emerged as a tumorigenic gene that triggers tumour metastasis and progression.^23^, ^24^ STAT4 was reported to activate IL8 transcription and the production of inflammatory mediators.^25^ STAT3 has also been reported to participate in and activate IL8‐regulated tumour angiogenesis.^26^ IL8, a member of the chemokine family, was proven to be involved in tumour angiogenesis and metastasis in BLCA and other malignant cancers.^27^, ^28^, ^29^ Currently, researchers have not determined whether STAT3/4 is involved in IL8‐mediated modulation of BLCA angiogenesis.

Here, we observed markedly upregulated OLCN expression in BLCA that was significantly associated with tumour angiogenesis in both patients with BLCA and murine xenograft models. Furthermore, OCLN overexpression enhanced the activity of the IL8/STAT3 signalling pathway by activating STAT4, ultimately contributing to tumour vascular remodelling and metastasis. Therefore, this study focused on the biological mechanism of OCLN in BLCA angiogenesis and provides potential targets for tumour treatment.

## 2 MATERIALS AND METHODS

### 2.1 Cell culture and reagents

BLCA cell lines, namely T24 (ATCC HTB‐4), 5637 (ATCC HTB‐9) were derived from laboratory retained cells.^30^ Human umbilical vein endothelial cells (EA.hy926) (ATCC CRL‐2922) were purchased from Conservation Genetics CAS Kunming Cell Bank and 293T (ATCC CRL‐11268) cells were obtained from ATCC. BLCA cell lines T24 and 5637 were cultured in RPMI‐1640 medium (Gibco, Carlsbad, CA, USA) containing 10% foetal bovine serum (FBS) (AusgeneX, Brisbane, Queensland, Australia). EA.hy926 cells were cultured in Dulbecco’s modified Eagle’s medium (DMEM)/F‐12 (Gibco, Carlsbad, CA, USA) supplemented with 10% FBS. 293T cells were cultured in DMEM (Gibco, Carlsbad, CA, USA) containing 10% FBS. All cell lines were incubated in a humidified incubator with 5% CO_2_ at 37°C.

### 2.2 Western blot assay

Cells were lysed in RIPA buffer on ice for 30 min, shaken 3 times every 10 min, and then centrifuged at 12 000 rpm for 15 min. The proteins in the cell supernatant (protein lysates) were separated on 8% to 12% SDS‐PAGE and then transferred to PVDF membranes (Millipore, Billerica, MA, USA). After blocking with 5% skim milk at room temperature for 1 h, the PVDF membranes were incubated with the primary antibody at 4°C overnight. The following antibodies were used: β‐Actin (2D4H5, mouse, ProteinTech, Wuhan, China, 1:5000), OCLN (ab216327, rabbit, Abcam, Cambridge, MA, USA, 1:1000), p‐STAT3 (#9145, rabbit, CST, Danvers, MA, USA, 1:1000), STAT3 (#9139, rabbit, CST, Danvers, MA, USA, 1:1000) and HSP90 (3F11C1, mouse, ProteinTech, Wuhan, China, 1:5000). The corresponding secondary antibodies were as follows: HRP‐conjugated horse anti‐mouse (7076, CST, Danvers, Massachusetts, USA, 1:2000) and goat anti‐rabbit (7074, CST, Danvers, Massachusetts, USA, 1:2000) secondary antibodies. β‐Actin and HSP90 were used as the internal controls.

### 2.3 Matrigel plug assay

BALB/c nude mice (male, 6–8 weeks) were randomly divided into 4 groups (*n *= 5 mice/group), and 2 × 10^6^ tumour cells were injected subcutaneously into the backs of mice. The cell suspension and 150 µl of Matrigel (#356231, Corning, NY, USA) were mixed at a ratio of 1:3. After two weeks, the Matrigel plugs were collected and photographed, followed by IHC staining and a pathological examination. All animal experimental procedures were approved by the Animal Experimental Ethics Committee of Sun Yat‐Sen University.

### 2.4 Plasmid and lentiviral infection

To generate T24 and 5637 OCLN knockdown cells, 293T cells were transduced with packaging plasmids psPAX2 and pMD2.G together with prepared pLKO.1‐OCLN shRNA or pLKO.1‐scrambled shRNA lentivirus. After transfection for 48 h, viral supernatants were collected and filtered, and then used to infect T24 and 5637 cells. Twenty‐four hours later, the cells were screened with puromycin (antpr‐1, InvivoGen)‐containing selection media (0.5 μg/ml) for 24 h. OCLN knockdown efficiency was measured by Western blotting and RT‐qPCR assays. The following shRNA sequences were used: shOCLN‐1 forward: 5′‐CCGGGCACCAAGCAATGACATATATCTCGAGATATATGTCATTGCTTGGTGCTTTTTG‐3′, reverse: 5′‐AATTCAAAAAGCACCAAGCAATGACATATATCTCGAGATATATGTCATTGCTTGGTGC‐3′; shOCLN‐2 forward: 5′‐CCGGGGATGACTATAGAGAAGAAAGCTCGAGCTTTCTTCTCTATAGTCATCCTTTTTG‐3′, reverse: 5′‐AATTCAAAAAGGATGACTATAGAGAAGAAAGCTCGAGCTTTCTTCTCTATAGTCATCC‐3′; and scrambled shRNA forward: 5′‐CCGGGCTAAACTCGTAATTCAACTTCTCGAGAAGTTGAATTACGAGTTTAGCTTTTTG‐3′, reverse: 5′‐AATTCAAAAAGCTAAACTCTAATTCAACTTCTCGAGAAGTTGAATTACGAGTTTAGC‐3′.

### 2.5 Plasmids and transient transfection procedures

The primer sequences for the Flag‐OCLN (NCBI Gene ID: 100506658) plasmid were as follows: forward: 5′‐TTTAAACTTAAGCTTGGTACCATGTCATCCAGGCCTCTTGA‐3′ and reverse: 5′‐GTCATCCTTGTAATCGAATTCTGTTTTCTGTCTATCATAGT‐3′. The HA‐STAT4 (NCBI Gene ID:6775) plasmid primer sequences were as follows: forward: 5′‐TTTAAACTTAAGCTTGGTACCGCCACCATGTCTCAGTGGAATCAAGTCCAAC‐3′ and reverse: 5′‐GACGTCGTATGGGTAGAATTCTTCAGCAGAATAAGGAGACTTCATT‐3′. Genes were amplified by reverse transcription PCR and cloned into the pcDNA3.1(+) vector. All sequences were verified to be correct. The relevant plasmids were transiently transfected into 5637, T24 and 293T cells using Lipofectamine™ 3000 (L3000015, Invitrogen, Carlsbad, CA, USA) according to the manufacturer’s instructions.

### 2.6 siRNAs and transient transfection procedures

All OCLN‐specific RNAs (siRNAs) and the negative control siRNA were designed by and purchased from RiboBio (stB0018520A/B‐1‐5, siN0000002‐1‐5, Guangzhou, China). The siRNAs were transfected into 293T cells using Lipofectamine™ 3000. 24 hours after the transfection of siRNAs, the cells were lysed for Western blotting or RT‐qPCR assays.

### 2.7 Tube formation assay

Matrigel (50 μl/well) was coated in each well of a 96‐well plate and incubated at 37°C for 30 min. After the polymerization of Matrigel, EA.hy926 cells (2 × 10^4^ cells/well) were resuspended in 100 μl of conditioned medium (CM), seeded on Matrigel and incubated at 37°C for 6–8 h. The cells were then stained with 1 μM Calcein‐AM (425201, Biolegend, San Diego, CA, USA) for 30 min and imaged with a fluorescence microscope (Nikon Corporation, Tokyo, Japan). The number of tubes was quantified in each image, and the total number of meshes and segments was calculated using ImageJ software.

### 2.8 Enzyme‐linked immunosorbent assay (ELISA)

Cells were transfected with the corresponding plasmids, and the supernatant was collected and centrifuged to completely remove cell debris after 48 h. A human IL8 ELISA kit (EK0413, BOSTER, Wuhan, China) was used to detect the IL8 concentration in CM according to the manufacturer’s instructions. Levels of the secreted protein were measured from a standard curve.

### 2.9 Clinical patients and immunohistochemistry (IHC) assay

All BLCA specimens (*n* = 120) were collected from the Fifth Affiliated Hospital of Sun Yat‐Sen University and were used after obtaining written consent from the patients. The collected tissues were first embedded in paraffin and cut into 4‐μm‐thick slices before the IHC assay. Sections were baked at 65°C for 3 h and then deparaffinized in xylene and alcohol. After dewaxing, the samples were washed with water and incubated with a citrate buffer solution (pH 9.0) in a pressure cooker for 2.5 min for antigen retrieval. Sections were treated with 3% hydrogen peroxide in methanol to block endogenous peroxidase activity and then incubated with goat serum for 30 min to block nonspecific staining. The sections were incubated with OCLN (ab216327, rabbit, Abcam, Cambridge, MA, USA, 1:100) and CD31 (ARG52748, rabbit, Arigobio, Hsinchu City, Taiwan, 1:100) primary antibodies followed by secondary antibodies. A biotinylated secondary antibody (PV‐9000, ZSGB‐BIO, Beijing, China) was applied, followed by an incubation with 3,3‐diaminobenzidine tetrahydrochloride (DAB). Finally, sections were counterstained with haematoxylin and sealed with neutral gum. Five fields were randomly selected from each sample to calculate expression. Scale bar = 100 µm. Staining/expression was scored as described in a previous study.^31^

### 2.10 Dual‐luciferase reporter assay

The IL8 promoter was used to drive firefly luciferase activity in the pGL3 luciferase vector. The pRL‐TK plasmid was cotransfected with the pGL3 vector in cells using Lipofectamine™ 3000 reagent to activate Renilla luciferase. Twenty‐four hours after transfection, the cells were harvested and washed with PBS, and then, the luciferase activity was measured using the Dual Luciferase Reporter Assay System (Promega, Madison, WI, USA) according to the manufacturer’s instructions. The IL8 promoter was cloned into the pGL3 vector with the following primers:

pGL3‐IL8 forward: ATTTCTCTATCGATA GGTACC TTCATTGTCCTGTACTTCCTGT and;

pGL3‐IL8 reverse: ACTTAGATCGCAGAT CTCGAG GTTTACACACAGTGAGATGGTT.

The firefly and Renilla luciferase activities were detected with a microplate reader. Firefly luciferase activity was normalized to Renilla luciferase activity.

### 2.11 Preparation of CM

5637 and T24 cells were seeded in 6‐well plates with RPMI‐1640 medium supplemented with 10% FBS. When cells reached confluence, they were washed with 1× PBS and cultured in serum‐free medium (1 ml/well). After an incubation for 4–6 h, the supernatant was replaced with fresh RPMI‐1640 medium containing 25% FBS and the STAT3 inhibitor Stattic (2 or 2.5 μM; #9983‐44‐9, MedChemExpress, NJ, USA). After 24 h, the medium was replaced with fresh RPMI‐1640 medium containing 10% FBS, and then the supernatant was collected after 24 h. After the cells were cultured with recombinant human IL8 (1 × 10^−4^ g/L; Peprotech, CI6217, Princeton Business Park, NJ, USA) and human antibody IL8 (5×10^−4^ g/L; Biotechne, MAB208‐SP, Minneapolis, MN, USA) for 24 h separately, they were switched to fresh RPMI‐1640 medium containing 10% FBS, and the supernatant was collected after 24 h.

### 2.12 Quantitative real‐time PCR assay

Total RNA was extracted from cells using TRIzol reagent (Invitrogen, Carlsbad, CA, USA), and then reverse transcribed into cDNAs using the Evo M‐MLV RT Kit (#AG11705, Accurate Biology, Changsha, China). Subsequently, cDNAs were amplified using a CFX96 real‐time PCR system (Bio‐Rad, Hercules, CA) and quantified with SYBR‐Green Pro Tap HS premix (#AG11701, Accurate Biology, Changsha, China). The sequences of the gene‐specific primers were as follows: h‐β‐Actin forward: 5′‐TCAAGATCATTGCTCCTCCTG‐3′, reverse: 5′‐CTGCTTGCTGATCCACATCTG‐3′; h‐OCLN forward: 5′‐ACTTCAGGCAGCCTCGTTAC‐3′, reverse: 5′‐CCTGATCCAGTCCTCCTCCA‐3′; h‐IL8 forward: 5′‐CACCGGAAGGAACCATCTCAC‐3′, reverse: 5′‐TGGTCCACTCTCAATCACTCTCAG‐3′; h‐STAT4 forward: 5′‐GGAAATTCGGCATCTGTTGGCC‐3′, reverse: 5′‐TTCTCTTTGGAAACACGACCTAACTGT‐3′; h‐ANG forward: ACTGTGTCCTCTTCCACCAC, reverse: GGATGTTTAGGGTCTTGCTTT; h‐MMP9 forward 5′‐AAGGCGCAGATGGTGGAT‐3′, reverse 5′‐TCAACTCACTCCGGGAACTC‐3′; and h‐IL18 forward: ATCAACCTCAGACCTTCCAG, reverse: GCATTATCTCTACAGTCAG. h‐β‐Actin was used as an internal control. The cycling conditions were 95°C for 30 s, followed by 40 cycles of 95°C for 5 s and 60°C for 30 s. The fold change in gene expression was calculated using the 2^−ΔΔCT^ method.

### 2.13 Statistical analysis

SPSS Standard version 25.0 (SPSS, Chicago, IL, USA) was applied for statistical analyses. All data were analysed and are expressed as the mean ± SD. Deviation from at least three independent experiments. *.01< *p* < .05; **.001< *p* < .01; ***p < .001. Student’s *t*‐test was used for comparisons of two independent datasets.

## 3 RESULTS

### 3.1 OCLN expression is upregulated and associated with the prognosis of patients with BLCA

**FIGURE 1:**
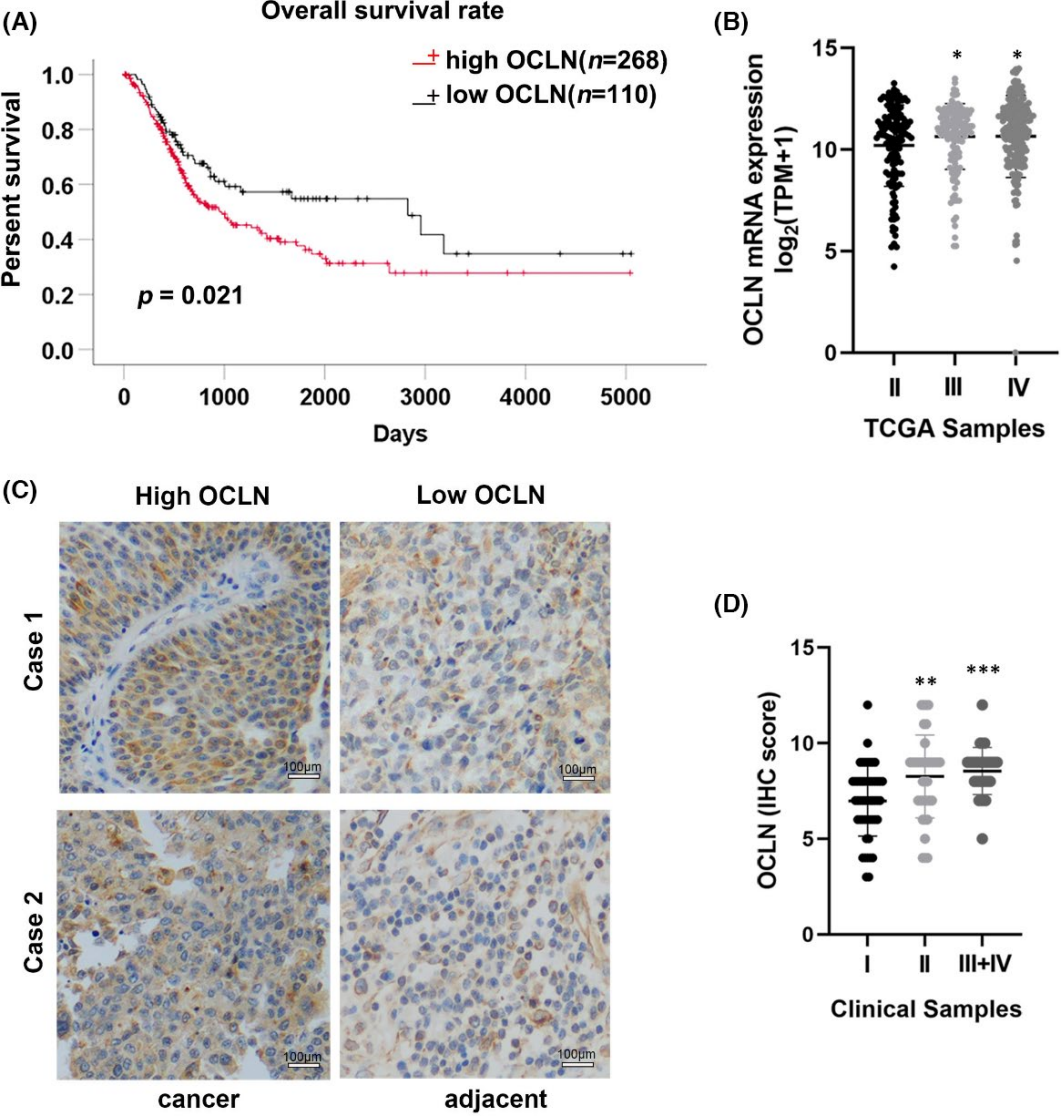
Occludin (OCLN) expression is associated with the prognosis of patients with bladder cancer (BLCA). (A) Kaplan–Meier plot of TCGA‐BLCA clinical datasets showed the overall survival rate of patients with OCLN^High^ (red line) and OCLN^Low^ (black line) expression (raw *p *= .021). (B) The TCGA‐BLCA clinical datasets showed the relationship between OCLN expression and BLCA neoplasm staging, and the number of stage I patients was too low to be counted. (C) IHC staining for OCLN expression in BLCA tissues and adjacent normal tissues. Scale bar = 100 µm. (D) The relationship between OCLN expression and BLCA neoplasm stages was determined in 120 clinical patients with BLCA. The results are shown as the mean ± SD. *.01< *p* < .05; **.001< *p* < .01; ***p < .001

We analysed the relationship of OCLN expression with the overall survival rate of patients with BLCA to determine whether OCLN is clinically associated with BLCA progression. The Kaplan–Meier plot indicated that patients in the Cancer Genome Atlas (TCGA)‐BLCA cohort with high OCLN expression had a lower survival rate than those with low OCLN expression (Figure 1A). Moreover, the statistical analysis of TCGA data revealed that OCLN expression was correlated with pathological stage (Figure 1B). To further verify the above results, we collected 120 bladder urothelial carcinoma specimens and evaluated OCLN staining. Consistent with the results obtained from the TCGA, IHC analysis showed higher OCLN expression in BLCA tumours than in adjacent tissues (Figure 1C), and OCLN expression was higher in patients with advanced tumours than in non‐advanced patients (Figure 1D). We next detected the correlations of the OCLN expression pattern with BLCA characteristics and risk factors. All these analyses indicated that the extent of invasion and clinical stages were associated with OCLN expression (Table 1). Collectively, these results indicated that OCLN expression is strongly associated with BLCA clinical features, and the protein may function as an oncogene in the development and progression of BLCA.

**TABLE 1:** Analysis of Occludin expression and clinicopathological features of patients with BLCA

Category	Numbers (*n *= 120)	Relative OCLN levels high low	*p* value
*Sex*			
Male	105	82 23	.867
Female	15	12 3	
*Age*			
≤65 years	48	40 8	.278
>65 years	72	54 18	
*Extent of invasion*			
T1 + T2	95	70 25	.016*
T3 + T4	25	24 1	
*Lymphatic metastasis*			
N0	116	90 26	.285
N1 + N2	4	4 0	
*Distant metastasis*			
M0	115	89 26	.230
M1	5	5 0	
*Clinical stages*			
I + II	90	65 25	.005*
III + IV	30	29 1	

* .01 < *p* < .05.

### 3.2 OCLN promotes BLCA angiogenesis in vitro and in vivo

**FIGURE 2:**
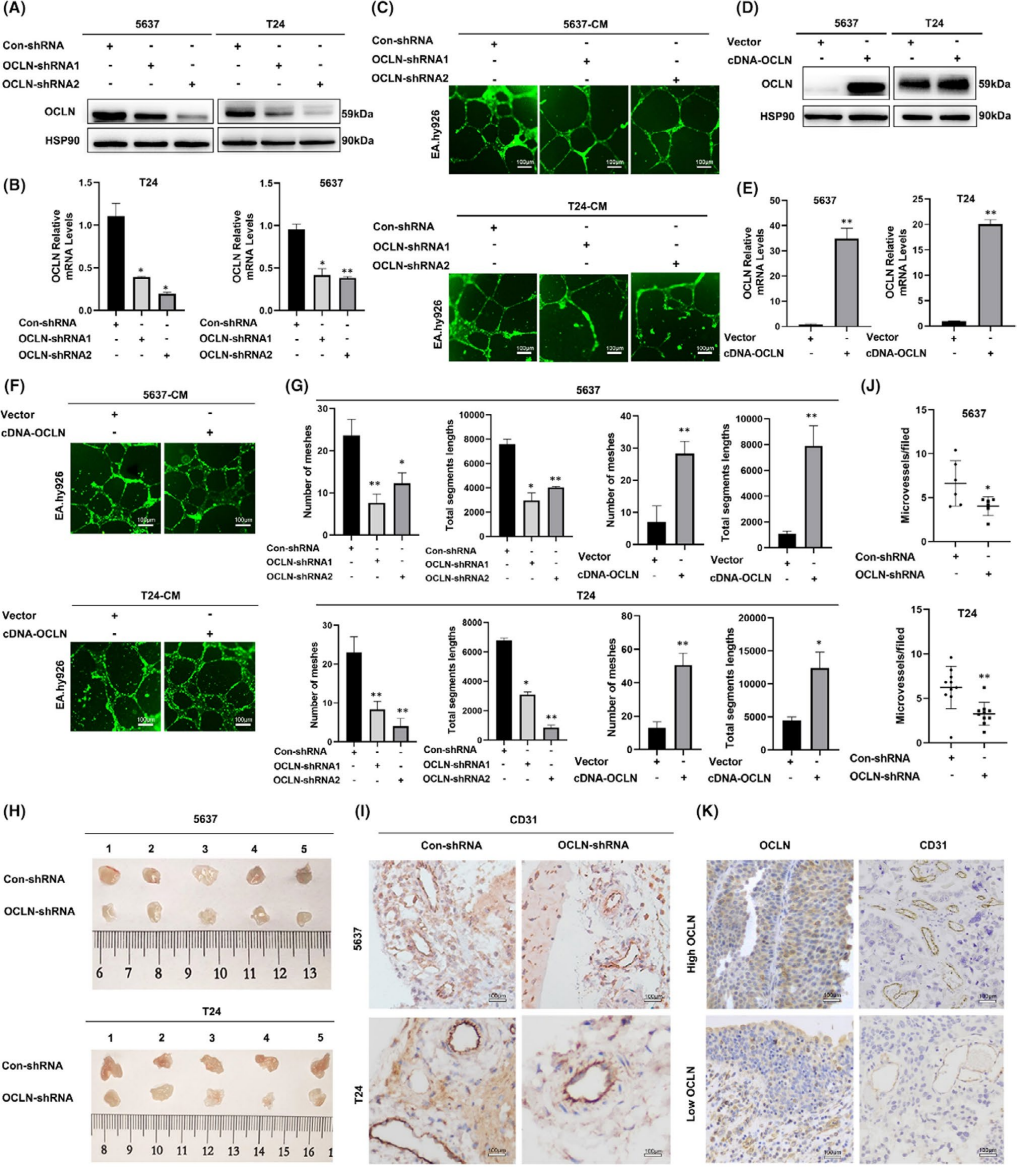
Occludin (OCLN) promotes tumour angiogenesis in vitro and in vivo. (a and b) The knockdown efficiency was confirmed by performing (A) Western blotting and (B) RT‐qPCR assays using 5637 and T24 cells. (C), Tube formation by EA.hy926 cells incubated with CM derived from 5637 and T24 OCLN‐silenced bladder cancer (BLCA) cells was assessed using staining with Calcein AM and imaging with a fluorescence microscope. Scale bar = 100 µm. (d and e) The OCLN plasmid was transfected into 5637 and T24 cells; the efficiency of overexpression was analysed using (D) Western blotting and (E) RT‐qPCR. (F) Tube formation by EA.hy926 cells incubated with CM derived from 5637 and T24 OCLN overexpressing cells was assessed using staining with Calcein AM and imaging with a fluorescence microscope. Scale bar = 100 µm. (G) The segment lengths were analysed, and the meshes were quantified using ImageJ software. (H) Matrigel plugs containing 5637 and T24 OCLN stable knockdown cells were removed from BALB/c nude mice. (I) CD31 staining in the indicated cells embedded in Matrigel plugs after growth in BALB/c nude mice. Scale bar = 100 µm. (J) The density of microvessels in Matrigel plugs from BALB/c nude mice injected with the indicated cells. (K) IHC staining showing CD31 levels in clinical patients with high‐/low‐grade BLCA. Scale bar = 100 µm. The results are shown as the mean ± SD. *.01 < *p* < .05; **.001 < *p* < .01

BLCA is a highly vascularized tumour, and we investigated whether OCLN was involved in the development of BLCA by regulating tumour angiogenesis. We constructed stable 5637 and T24 OCLN knockdown BLCA cell lines using lentiviruses to verify this hypothesis, and the knockdown efficiency was confirmed by Western blotting and RT‐qPCR assays (Figure 2A,B). CM from 5637 and T24 OCLN knockdown cells was collected to assess the role of OCLN in regulating endothelial cell angiogenesis. Decreased tube formation was observed in EA.hy926 cells cultured with CM derived from OCLN‐silenced BLCA cells (Figure 2C). Conversely, the number of cell intersections was obviously increased when EA.hy926 cells were incubated with CM derived from OCLN‐overexpressing BLCA cells (Figure 2D–F). Quantification of the number of meshes and total segment lengths further confirmed these results (Figure 2G). Next, we performed an in‐depth analysis of the role of OCLN in regulating BLCA angiogenesis in vivo. A Matrigel plug assay was performed by subcutaneously injecting Matrigel containing 5637 and T24 stable OCLN knockdown cells into BALB/c nude mice. The surface of plugs presented red staining, indicating that many new blood vessels had been formed in the plugs, while OCLN‐silenced plugs showed significantly fewer blood vessels (Figure 2H). More importantly, CD31 staining of plugs revealed that OCLN knockdown substantially downregulated OCLN expression and decreased the density of microvessels (Figure 2I,J). We also detected CD31 levels in clinical patients with BLCA carcinoma and paraneoplastic tissue and found that CD31 was expressed at higher levels in high‐grade BLCA samples than in low‐grade samples (Figure 2K). Furthermore, OCLN expression was positively correlated with angiogenesis phenotypes (Table 2). All of these findings verified that OCLN regulates BLCA angiogenesis. Taken together, OCLN promotes BLCA angiogenesis in vitro and in vivo.

**TABLE 2:** Correlation between CD31 and Occludin expression

Category	Numbers (*n* = 120)	Relative of OCLN levels High Low	*p value*
*CD31 scores (median = 27.5)*			
≤27.5	66	47 19	.036*
>27.5	54	47 7	

* .01 < *p* < .05.

### 3.3 OCLN mediated BLCA angiogenesis by regulating IL8 expression

**FIGURE 3:**
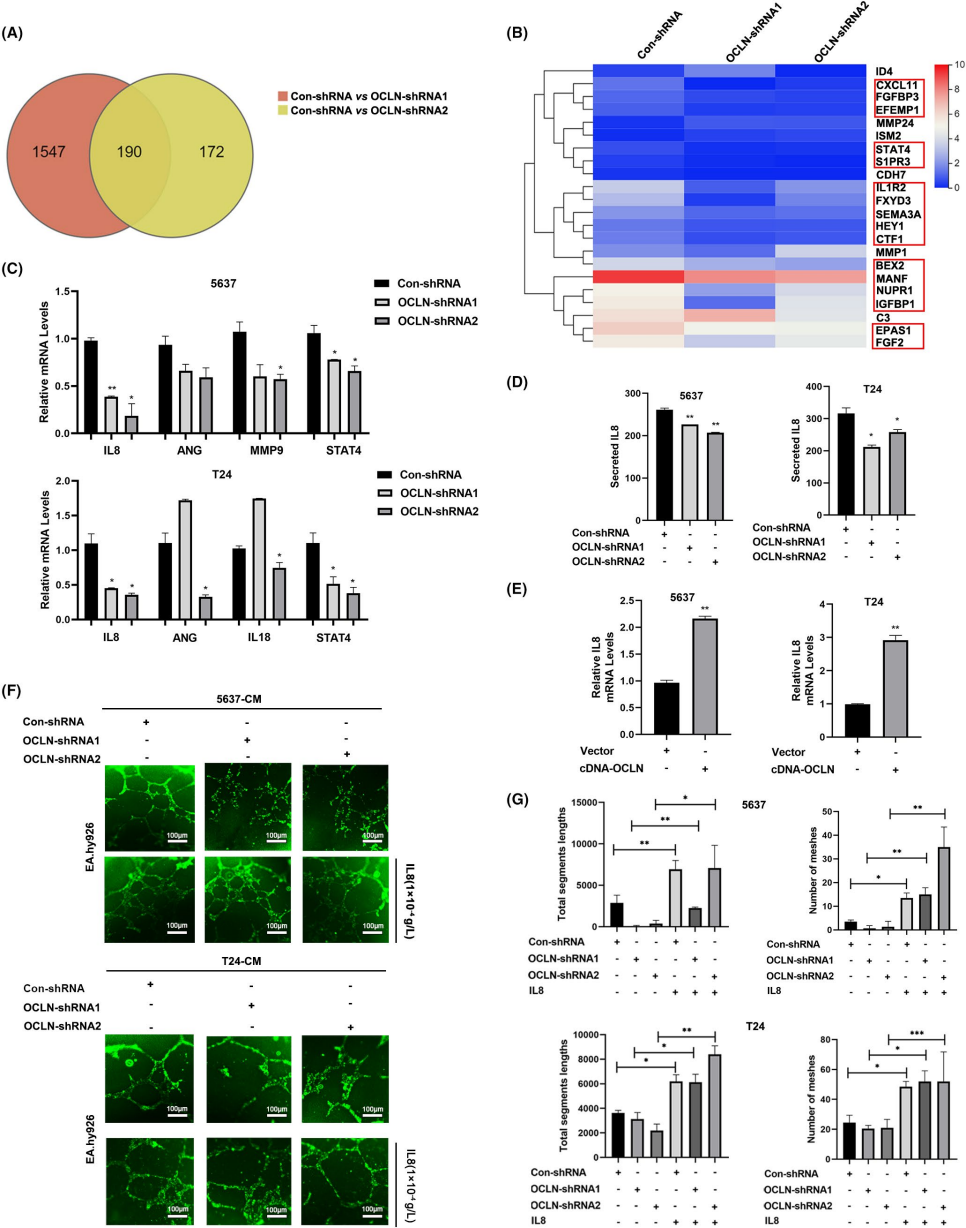
Occludin (OCLN) mediates bladder cancer (BLCA) angiogenesis by regulating IL8 expression. (A) Venn diagram showing the differentially expressed genes (DEGs) in the two T24 OCLN knockdown groups compared with the control groups (fold change ≥1, FDR < 0.1, *p* < .05). (B) Heatmap of the RNA sequencing analysis showing the relative levels of proangiogenic factors. Columns represent probe sets, and rows represent samples receiving the indicated treatments. (C) The relative mRNA levels of proangiogenic factors were detected in control and OCLN shRNA transfected 5637 and T24 cells. (D) The relative IL8 levels in control and OCLN shRNA‐transfected 5637 and T24 cells were measured using an ELISA (pg/ml). (E) The relative IL8 mRNA levels were detected in 5637 and T24 cells following transfection with the vector or OCLN plasmid. (F) Tube formation by EA.hy926 cells cultured with CM derived from 5637 and T24 OCLN‐silenced BLCA cells. IL8 was added, and the cells were stained with Calcein AM and then imaged with a fluorescence microscope. Scale bar = 100 µm. (G) The segment lengths were analysed, and the meshes were quantified using ImageJ software. The results are shown as the mean ± SD. *.01 < *p* < .05; **.001 < *p* < .01; ***p < .001

We performed RNA sequencing to identify changes in gene expression in T24 OCLN‐silenced cells and to further investigate the mechanism by which OCLN regulates angiogenesis. The two T24 OCLN‐shRNA groups presented 1547 and 172 downregulated genes, respectively, and 190 genes were common to the two groups (Figure 3A). We performed an enrichment analysis of these downregulated genes and found that they were enriched in pathways such as protein processing in the endoplasmic reticulum, cytokine‐cytokine receptor interactions, and abnormal transcriptional regulation in the cancer pathway (Figure S1a). More interestingly, the functional annotation analysis identified many extracellular gene sets, including those related to exosomes, extracellular spaces and the extracellular matrix (Figure S1b). According to previous reports, tumour cells generally secrete large amounts of angiogenic factors into the extracellular matrix, promoting the formation of new blood vessels and tumour metastasis,^32^ which provides further support for the regulation of tumour angiogenesis by OCLN in BLCA. As expected, 22 of these genes were associated with the promotion of angiogenesis, and 16 of these genes expression were decreased in OCLN‐silenced cells compared with control cells (Figure 3B), suggesting that OCLN may regulate BLCA angiogenesis by regulating genes associated with angiogenesis. In addition, we performed an RT‐qPCR assay to detect the expression of angiogenic factors after OCLN knockdown; IL8 was the most substantially downregulated gene among them (Figure 3C), and this finding was further validated by ELISA in OCLN knockdown BLCA cells (Figure 3D). Consistently, OCLN overexpression increased IL8 expression (Figure 3E). As the chemokine, IL8 has been shown to induce angiogenesis in multiple vascular‐rich malignancies.^33^ We restored IL8 expression in OCLN‐silenced cells and collected CM to monitor the tube formation of endothelial cells and further confirm whether IL8 was involved in the mechanism by which OCLN regulates BLCA angiogenesis. IL8 enhanced the tube formation of EA.hy926 cells induced by the downregulation of OCLN (Figure 3F,G). Collectively, IL8 is involved in OCLN‐mediated regulation of BLCA angiogenesis.

### 3.4 IL8 is involved in the process of OCLN‐mediated STAT3 angiogenesis in BLCA

**FIGURE 4:**
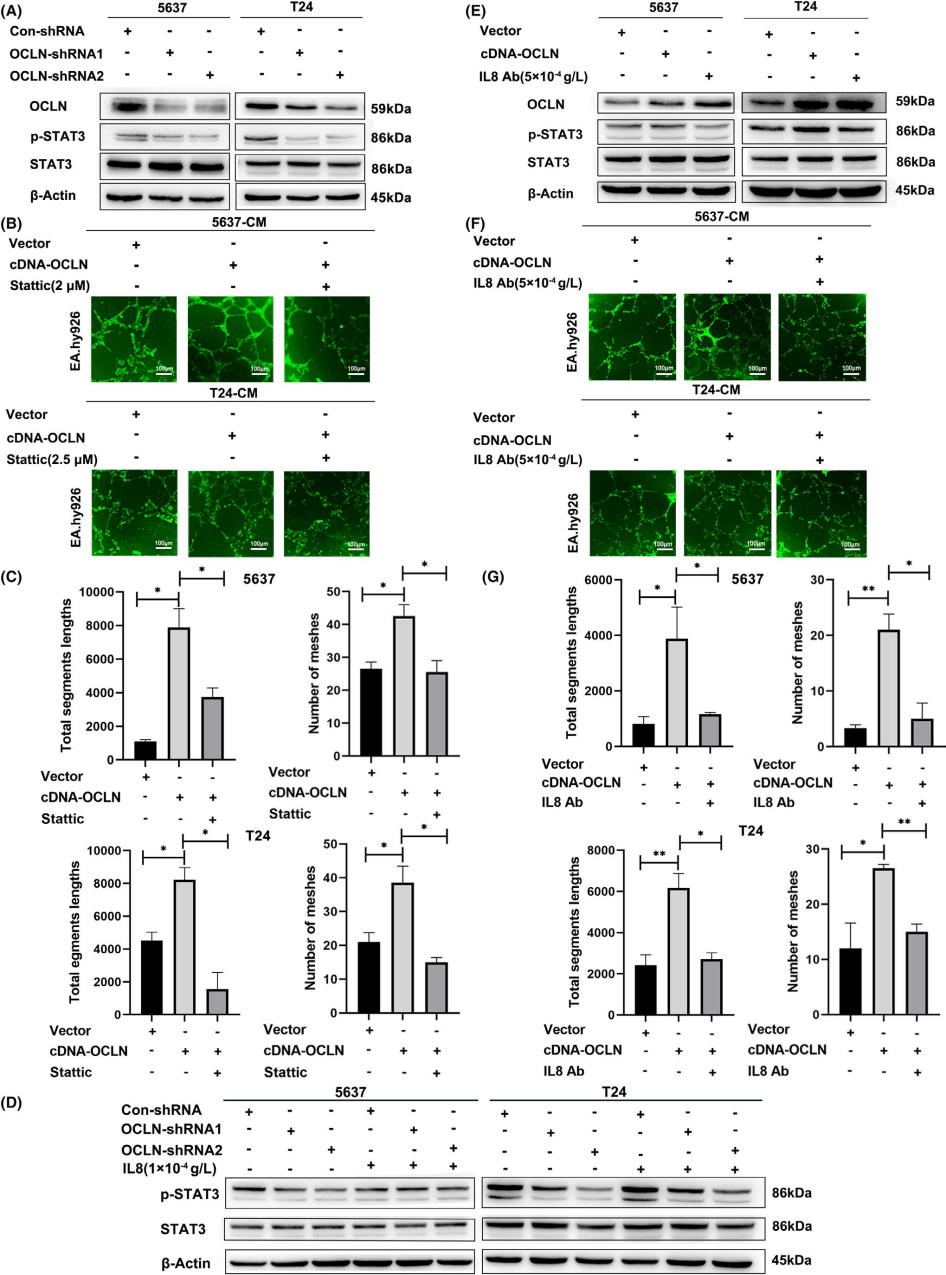
IL8/ STAT3 is involved in the process of Occludin (OCLN)‐mediated angiogenesis in bladder cancer (BLCA). (A) p‐STAT3 and STAT3 protein levels were detected in OCLN knockdown 5637 and T24 cells. (B) 5637 and T24 cells were transfected with the OCLN plasmid and treated with or without the STAT3 inhibitor Stattic, and tube formation by EA.hy926 cells incubated with CM derived from the indicated cells was assessed by performing staining with Calcein AM and imagining using a fluorescence microscope. Scale bar = 100 µm. (C) The segment lengths in these images were analysed, and the meshes were quantified using ImageJ software. D, p‐STAT3 and STAT3 protein levels were detected in OCLN knockdown 5637 and T24 cells after IL8 supplementation. (E) p‐STAT3 and STAT3 protein levels were detected in OCLN‐overexpressing 5637 and T24 cells after blocking IL8. (F) 5637 and T24 cells were transfected with the OCLN plasmid or cultured with the IL8‐neutralizing antibody; tube formation by EA.hy926 cells incubated with CM derived from the indicated cells was assessed using staining with Calcein AM and imaging with a fluorescence microscope. Scale bar = 100 µm. G, The segment lengths in these images were analysed, and the meshes were quantified using ImageJ software. The results are shown as the mean ± SD. *.01< *p* < .05; **.001< *p* < .01

We next explored the detailed mechanism by which OCLN regulates IL8 expression in BLCA angiogenesis. Previous studies have shown that STAT3 is involved in vascular remodelling and progression in breast cancer^34^; however, researchers have not reported whether STAT3 participates in OCLN‐mediated regulation of BLCA angiogenesis. We first detected the expression of proteins in the STAT3 pathway after OCLN knockdown, and p‐STAT3 levels were significantly decreased in 5637 and T24 OCLN‐silenced cells compared control cells, while total STAT3 levels remained unchanged (Figure 4A). We then used the STAT3 inhibitor Stattic to assess the role of STAT3 in OCLN‐mediated regulation of BLCA angiogenesis. Tube formation assays confirmed that Stattic blocked the proangiogenic effect of OCLN overexpression (Figure 4B,C), indicating that STAT3 was involved in OCLN‐modified modulation of tumour angiogenesis in BLCA. In addition, we found that IL8 supplement restored p‐STAT3 expression caused by the downregulation of OCLN (Figure 4D), suggesting that IL8 may participate in OCLN‐mediated regulation of the STAT3 pathway in BLCA angiogenesis. Conversely, the IL8‐neutralizing antibody blocked the increase in p‐STAT3 levels and inhibited tube formation caused by OCLN overexpression (Figure 4E–G), further confirming the role of IL8 in OCLN‐mediated STAT3‐induced angiogenesis. Taken together, these results indicated that OCLN controls tumour angiogenesis by regulating p‐STAT3 levels in BLCA cells through effects on IL8 expression.

### 3.5 OCLN regulates the IL8/STAT3 pathway to mediate BLCA angiogenesis through STAT4

**FIGURE 5:**
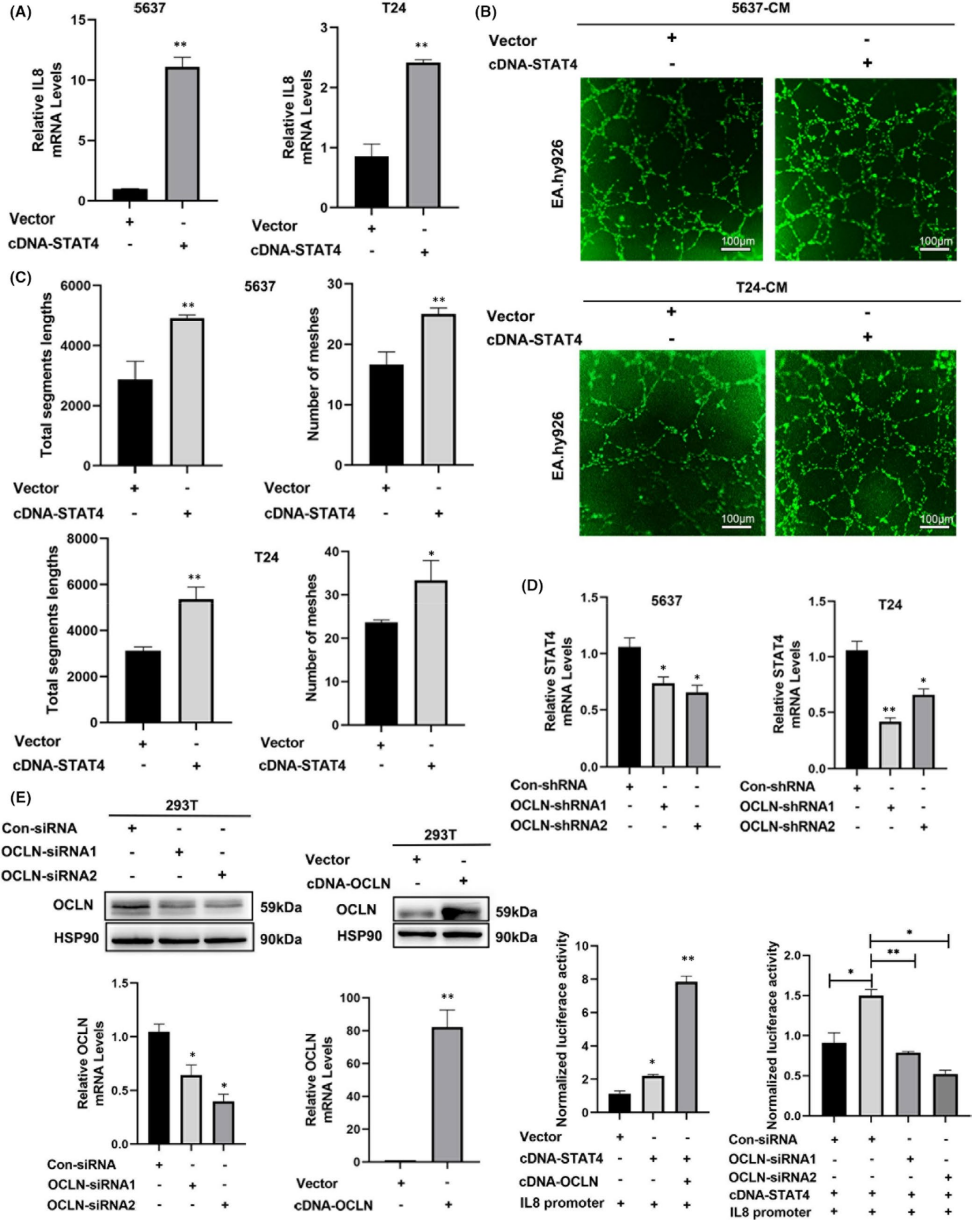
STAT4 is involved in the mechanism by which Occludin (OCLN) regulates IL8/STAT3 signalling to mediate bladder cancer angiogenesis. (A) Relative IL8 mRNA levels were detected in control and STAT4‐overexpressing 5637 and T24 cells using RT‐qPCR. (B) The STAT4 plasmid or the indicated vector was transfected into 5637 and T24 cells, and tube formation by EA.hy926 cells incubated with CM derived from the indicated cells was assessed using staining with Calcein AM and imaging with a fluorescence microscope. Scale bar = 100 µm. (C) The segment lengths were analysed, and the meshes were quantified using ImageJ software. (D) The relative STAT4 mRNA levels were detected in control and OCLN shRNA‐transfected 5637 and T24 cells. (E) Cells were transfected with the indicated siRNAs or plasmids for 24 h. The relative knockdown and overexpression efficiency was determined using Western blotting and RT‐qPCR. (F) 293T cells were cotransfected with the indicated plasmids for 24 h, and the relative activity of the IL8 promoter was evaluated by performing a dual luciferase reporter assay. The results are shown as the mean ± SD. *.01< *p* < .05; **.001< *p* < .01

In the enrichment analysis of proangiogenic genes in OCLN knockdown cells shown in Figure 3B, we identified another significantly downregulated STAT family protein, STAT4, which has been reported to be involved in the occurrence and development of multiple tumours and tissue angiogenesis.^35^, ^36^, ^37^ In addition, STAT4 has been reported to promote angiogenesis in pancreatic cancer by inducing IL8 transcription.^38^ However, researchers have not yet clarified the potential association between STAT4 and IL8 in BLCA angiogenesis. First, we detected IL8 expression in 5637 and T24 cells overexpressing STAT4, and the results indicated that STAT4 overexpression upregulated IL8 levels (Figure 5A). In addition, tube formation assays indicated an obvious increase in the number of cell intersections when EA.hy926 cells were incubated with CM derived from STAT4 overexpressing BLCA cells (Figure 5B,C), suggesting that STAT4 participated in BLCA angiogenesis may partially by regulating IL8 expression. Furthermore, we again confirmed the inhibitory effect of STAT4 on 5637 and T24 OCLN knockdown cells (Figure 5D), and found that OCLN promotes IL8 expression through STAT4 in BLCA cells. We focused on the role of STAT4 as a transcription factor in tumour angiogenesis and sought to explore whether OCLN mediated the transcription of IL8 via STAT4 as an approach to further verify the role of STAT4 in OCLN‐mediated regulation of IL8 expression in BLCA angiogenesis. We used transient transfection to verify the knockdown and overexpression efficiency of OCLN in 293T cells (Figure 5E). In addition, a dual luciferase reporter gene assay indicated that the STAT4‐mediated transcription of IL8 was significantly increased upon the overexpression of OCLN. Conversely, OCLN knockdown significantly decreased STAT4‐induced IL8 transcription (Figure 5F). Collectively, these results indicate that OCLN regulates BLCA angiogenesis through the transcriptional regulation of IL8 in a STAT4‐dependent manner.

## 4 DISCUSSION

BLCA is the most common urothelial tumour, accounting for approximately 430 000 newly diagnosed cases and 170 000 deaths worldwide annually.^39^ The mechanism underlying the malignant development of tumours is currently unclear. Indeed, some studies have shown that abnormal neovascularization is an important step in cancer progression and is a mechanistically attractive target for the treatment of tumors.^32^, ^40^ Here, we first proposed a role for OCLN in regulating BLCA angiogenesis. In our study, OCLN was overexpressed in BLCA cells and patients, and it may represent a tumorigenic factor, especially in the promotion of BLCA angiogenesis. Phenotypic experiments showed that OCLN expression promoted BLCA vascular tube formation in vivo and in vitro. Mechanistically, OCLN knockdown inhibited IL8 expression and then reduced p‐STAT3 levels to interrupt tumour angiogenesis. Furthermore, OCLN promoted the transcription of IL8 through STAT4, ultimately contributing to tumour vascular remodelling and BLCA progression.

Tumour neovascularization is essential for the metastasis of some malignant tumours, and various proangiogenic factors are involved in tumour progression, including angiopoietin (Ang), platelet‐derived growth factor (PDGF), basic fibroblast growth factor (bFGF), vascular endothelial growth factor (VEGF), IL8 and matrix metalloproteinases (MMPs).^41^ In the present study, OCLN was expressed at high levels and correlated strongly with the TNM stage in patients with BLCA (Figure 1). Because tumour development is based on tumour angiogenesis, which provides essential nutrients for tumour growth, we knocked down and overexpressed OCLN to detect the function of OCLN. OCLN exerted a proangiogenic effect on BLCA cells and regulated tube formation by endothelial cells cultured with CM derived from 5637 and T24 cells by altering IL8 levels (Figures 2C,F,G and 3F,G), indicating that OCLN promotes tumour neovascularization through IL8. Moreover, the RNA sequencing analysis revealed that OCLN‐silenced cells exhibited downregulation of proangiogenic factors and IL8 (Figure 3B–E), further supporting the hypothesis that OCLN mediates tumorigenesis by regulating the expression and secretion of angiogenic factors. Another tight junction protein, TJP1, was also reported to promote vasculature remodelling in bladder cancer.^30^ Although this report is the first to correlate OCLN and IL8 expression in BLCA angiogenesis, independent studies have revealed an association between IL8 and abnormal neovascularization properties. IL8 overexpression correlates with the TNM stage, tumour progression and recurrence in multiple cancers.^42^ IL8 produced by tumour cells has been reported to stimulate both tumour and stromal cells to release angiogenesis‐related factors, such as MMP2/9, thus promoting tumour growth. MMP2/9 activation subsequently increases the invasion of the host stroma by tumour cells, ultimately contributing to tumour angiogenesis and metastasis.^43^ Furthermore, our findings are consistent with those of studies reporting the role of IL8 in BLCA angiogenesis. Currently, antiangiogenic drugs are still one of the key targeted therapeutic strategies for many malignant cancers, including BLCA, which is highly vascularized. As shown in a previous study, antivascular drugs affect tumour development by regulating the expression of tight junction proteins,^44^ indicating an effect of tight junction proteins on the function of antiangiogenic drugs. Treatment with the proper dose bevacizumab increases claudin‐5 (CLDN5) expression to decrease tumour invasion and metastatic potential.^45^ In addition, a derivative of sorafenib reduces the activation of VEGF/VEGFR related to angiogenesis by increasing the ZO‐1 expression in HUVECs.^46^ As IL8 may play a role in OCLN‐mediated regulation of BLCA angiogenesis, further study is needed to determine if antiangiogenic drugs synergize with OCLN or IL8 inhibitors to regulate BLCA angiogenesis and to determine the specific mechanism.

According to a previous study, IL8 is involved in transcriptional regulation by activated NFκB,^47^ activating transcription factor 2 (ATF2), JUN, MAPK^48^ and other transcription factors; these transcription factors have also been reported to regulate tumour angiogenesis. However, our results indicated that OCLN downregulation did not alter the expression of the aforementioned factors except for STAT4, according to the RNA sequencing and RT‐qPCR results (Figures 3C and 5D). In addition, STAT4 promoted tube formation by endothelial cells cultured with CM derived from BLCA cells (Figure 5B,C), moreover, OCLN mediated IL8 transcription through STAT4 (Figure 5F), further confirming the role of STAT4 in BLCA angiogenesis. In addition, we showed that STAT3 participates in OCLN‐mediated regulation of tumour angiogenesis in BLCA (Figure 4B,C). OCLN altered p‐STAT3 levels but not total STAT3 levels (Figure 4A), potentially because STAT3 phosphorylation and transport into the nucleus is involved in the regulation of STAT4 transcriptional activity by OCLN. JAK phosphorylation is required for STAT4 activation.^49^, ^50^ Most likely, the regulation of these signalling pathways and subsequent transcription mainly depends on the posttranslational modifications, especially phosphorylation. Therefore, studies focusing on changes in the phosphorylation of proteins in STAT‐related pathways will be very important to identify the key factors contributing to OCLN‐regulated BLCA angiogenesis.

In summary, we discovered that OCLN expression is strongly correlated with the TNM stage and survival rate of patients with BLCA. In pathological samples and mouse xenograft models, OCLN was positively correlated with tumour angiogenesis. Importantly, OCLN regulated vascular remodelling in BLCA by regulating the IL8/STAT3 pathway through the induction of STAT4 expression. Collectively, these results indicate great potential for OCLN as a multipotent therapeutic target to inhibit tumour angiogenesis and progression in BLCA. As tumour angiogenesis plays an important role in the occurrence, development and metastasis of many tumours, our study expands the in‐depth knowledge of the possible effects of OCLN and proposes the basic principle of targeting OCLN to suppress angiogenesis in the treatment of metastatic tumours.

## CONFLICT OF INTEREST

All authors agree to submit the article and have no conflict of interest.

## AUTHOR CONTRIBUTIONS

**Fan Yang:** Data curation (equal); Investigation (equal); Writing – original draft (equal). **Xue‐Qi Liu:** Investigation (equal); Writing – original draft (equal). **Jian‐Zhong He:** Software (equal). **Shi‐Ping Xian:** Software (equal). **Peng‐Fei Yang:** Software (equal). **Zhi‐Ying Mai:** Software (equal). **Miao Li:** Funding acquisition (equal); Supervision (equal). **Ye Liu:** Funding acquisition (equal); Supervision (equal). **Xing‐Ding Zhang:** Funding acquisition (equal); Supervision (equal).

## Supporting information

Fig S1

## Data Availability

The datasets analysed during the current study are available from the corresponding author on reasonable request.

## References

[1] Dobruch J , Daneshmand S , Fisch M , et al. Gender and bladder cancer: a collaborative review of etiology, biology, and outcomes. Eur Urol. 2016;69(2):300‐310. 26346676 10.1016/j.eururo.2015.08.037

[2] Seidl C . Targets for therapy of bladder cancer. Semin Nucl Med. 2020;50(2):162‐170. 32172801 10.1053/j.semnuclmed.2020.02.006

[3] Nowak‐Sliwinska P , Alitalo K , Allen E , et al. Consensus guidelines for the use and interpretation of angiogenesis assays. Angiogenesis. 2018;21(3):425‐532. 29766399 10.1007/s10456-018-9613-x PMC6237663

[4] Ramjiawan RR , Griffioen AW , Duda DG . Anti‐angiogenesis for cancer revisited: is there a role for combinations with immunotherapy? Angiogenesis. 2017;20(2):185‐204. 28361267 10.1007/s10456-017-9552-y PMC5439974

[5] Popper HH . Progression and metastasis of lung cancer. Cancer Metastasis Rev. 2016;35(1):75‐91. 27018053 10.1007/s10555-016-9618-0 PMC4821869

[6] Lugano R , Ramachandran M , Dimberg A . Tumor angiogenesis: causes, consequences, challenges and opportunities. Cell Mol Life Sci. 2020;77(9):1745‐1770. 31690961 10.1007/s00018-019-03351-7 PMC7190605

[7] Wu S , Ou T , Xing N , et al. Whole‐genome sequencing identifies ADGRG6 enhancer mutations and FRS2 duplications as angiogenesis‐related drivers in bladder cancer. Nat Commun. 2019;10(1):720. 30755618 10.1038/s41467-019-08576-5 PMC6372626

[8] Wigner P , Grebowski R , Bijak M , Saluk‐Bijak J , Szemraj J . The interplay between oxidative stress, inflammation and angiogenesis in bladder cancer development. Int J Mol Sci. 2021;22(9):4483. 33923108 10.3390/ijms22094483 PMC8123426

[9] Saito AC , Higashi T , Fukazawa Y , et al. Occludin and tricellulin facilitate formation of anastomosing tight‐junction strand network to improve barrier function. Mol Biol Cell. 2021;32(8):722‐738. 33566640 10.1091/mbc.E20-07-0464 PMC8108510

[10] Wang M , Liu Y , Qian X , Wei N , Tang Y , Yang J . Downregulation of occludin affects the proliferation, apoptosis and metastatic properties of human lung carcinoma. Oncol Rep. 2018;40(1):454‐462. 29750300 10.3892/or.2018.6408

[11] Shi S , Cheng J , Zhang C , et al. Peripheral blood occludin level as a biomarker for perioperative cerebral edema in patients with brain tumors. Dis Markers. 2020;2020:8813535. 32884584 10.1155/2020/8813535 PMC7455817

[12] Wang K , Xu C , Li W , Ding L . Emerging clinical significance of claudin‐7 in colorectal cancer: a review. Cancer Manag Res. 2018;10:3741‐3752. 30288105 10.2147/CMAR.S175383 PMC6159786

[13] Lauko A , Mu Z , Gutmann DH , Naik UP , Lathia JD . Junctional adhesion molecules in cancer: a paradigm for the diverse functions of cell‐cell interactions in tumor progression. Cancer Res. 2020;80(22):4878‐4885. 32816855 10.1158/0008-5472.CAN-20-1829 PMC7669553

[14] Tornavaca O , Chia M , Dufton N , et al. ZO‐1 controls endothelial adherens junctions, cell‐cell tension, angiogenesis, and barrier formation. J Cell Biol. 2015;208(6):821‐838. 25753039 10.1083/jcb.201404140 PMC4362456

[15] Kanayasu‐Toyoda T , Ishii‐Watabe A , Kikuchi Y , et al. Occludin as a functional marker of vascular endothelial cells on tube‐forming activity. J Cell Physiol. 2018;233(2):1700‐1711. 28681912 10.1002/jcp.26082

[16] Schmitt M , Horbach A , Kubitz R , Frilling A , Haussinger D . Disruption of hepatocellular tight junctions by vascular endothelial growth factor (VEGF): a novel mechanism for tumor invasion. J Hepatol. 2004;41(2):274‐283. 15288477 10.1016/j.jhep.2004.04.035

[17] Liu X , Dreffs A , Díaz‐Coránguez M , et al. Occludin S490 phosphorylation regulates vascular endothelial growth factor‐induced retinal neovascularization. Am J Pathol. 2016;186(9):2486‐2499. 27423695 10.1016/j.ajpath.2016.04.018 PMC5012506

[18] Zeng Z , Li Y , Pan Y , et al. Cancer‐derived exosomal miR‐25‐3p promotes pre‐metastatic niche formation by inducing vascular permeability and angiogenesis. Nat Commun. 2018;9(1):5395. 30568162 10.1038/s41467-018-07810-w PMC6300604

[19] Pencik J , Pham HTT , Schmoellerl J , et al. JAK‐STAT signaling in cancer: from cytokines to non‐coding genome. Cytokine. 2016;87:26‐36. 27349799 10.1016/j.cyto.2016.06.017 PMC6059362

[20] Korac‐Prlic J , Degoricija M , Vilović K , et al. Targeting Stat3 signaling impairs the progression of bladder cancer in a mouse model. Cancer Lett. 2020;490:89‐99. 32659249 10.1016/j.canlet.2020.06.018

[21] Hindupur SV , Schmid SC , Koch JA , et al. STAT3/5 inhibitors suppress proliferation in bladder cancer and enhance oncolytic adenovirus therapy. Int J Mol Sci. 2020;21(3):1106. 10.3390/ijms21031106 PMC7043223 32046095

[22] Zhang W , Mao S , Shi D , et al. MicroRNA‐153 decreases tryptophan catabolism and inhibits angiogenesis in bladder cancer by targeting indoleamine 2,3‐dioxygenase 1. Front Oncol. 2019;9:619. 31355138 10.3389/fonc.2019.00619 PMC6636202

[23] Zhao L , Ji G , Le X , et al. An integrated analysis identifies STAT4 as a key regulator of ovarian cancer metastasis. Oncogene. 2017;36(24):3384‐3396. 28114283 10.1038/onc.2016.487

[24] Liu S , Li L , Zhang Y , et al. The oncoprotein HBXIP uses two pathways to up‐regulate S100A4 in promotion of growth and migration of breast cancer cells. J Biol Chem. 2012;287(36):30228‐30239. 22740693 10.1074/jbc.M112.343947 PMC3436276

[25] Nguyen HN , Noss EH , Mizoguchi F , et al. Autocrine loop involving IL‐6 family member LIF, LIF receptor, and STAT4 drives sustained fibroblast production of inflammatory mediators. Immunity. 2017;46(2):220‐232. 28228280 10.1016/j.immuni.2017.01.004 PMC5567864

[26] Jin G , Yang Y , Liu K , et al. Combination curcumin and (−)‐epigallocatechin‐3‐gallate inhibits colorectal carcinoma microenvironment‐induced angiogenesis by JAK/STAT3/IL‐8 pathway. Oncogenesis. 2017;6(10):e384. 28967875 10.1038/oncsis.2017.84 PMC5668882

[27] Chung HW , Lim JB . High‐mobility group box‐1 contributes tumor angiogenesis under interleukin‐8 mediation during gastric cancer progression. Cancer Sci. 2017;108(8):1594‐1601. 28574630 10.1111/cas.13288 PMC5543560

[28] Lesage J , Suarez‐Carmona M , Neyrinck‐Leglantier D , et al. Zonula occludens‐1/NF‐kappaB/CXCL8: a new regulatory axis for tumor angiogenesis. FASEB J. 2017;31(4):1678‐1688. 28057697 10.1096/fj.201600890R

[29] Karashima T , Sweeney P , Kamat A , Huang S , Dinney C . Nuclear factor‐kappaB mediates angiogenesis and metastasis of human bladder cancer through the regulation of interleukin‐8. Clin Cancer Res. 2003;9(7):2786‐2797. 12855659

[30] Liu X‐Q , Shao X‐R , Liu YE , et al. Tight junction protein 1 promotes vasculature remodeling via regulating USP2/TWIST1 in bladder cancer. Oncogene. 2022;41:502‐514. 34782718 10.1038/s41388-021-02112-w

[31] Zhang BO , Chan S‐H , Liu X‐Q , et al. Targeting hexokinase 2 increases the sensitivity of oxaliplatin by Twist1 in colorectal cancer. J Cell Mol Med. 2021;25(18):8836‐8849. 34378321 10.1111/jcmm.16842 PMC8435428

[32] Viallard C , Larrivee B . Tumor angiogenesis and vascular normalization: alternative therapeutic targets. Angiogenesis. 2017;20(4):409‐426. 28660302 10.1007/s10456-017-9562-9

[33] Imafuji H , Matsuo Y , Ueda G , et al. Acquisition of gemcitabine resistance enhances angiogenesis via upregulation of IL8 production in pancreatic cancer. Oncol Rep. 2019;41(6):3508‐3516. 31002348 10.3892/or.2019.7105

[34] Banerjee K , Resat H . Constitutive activation of STAT3 in breast cancer cells: a review. Int J Cancer. 2016;138(11):2570‐2578. 26559373 10.1002/ijc.29923 PMC4801660

[35] Li Y , Wang J , Chen W , Chen X , Wang J . Overexpression of STAT4 under hypoxia promotes EMT through miR‐200a/STAT4 signal pathway. Life Sci. 2021;273:e119263. 10.1016/j.lfs.2021.119263 33636177

[36] Li S , Sheng B , Zhao M , Shen Q , Zhu H , Zhu X . The prognostic values of signal transducers activators of transcription family in ovarian cancer. Biosci Rep. 2017;37(4):BSR20170650. 28536310 10.1042/BSR20170650 PMC5518537

[37] Shao J , Fan G , Yin X , et al. A novel transthyretin/STAT4/miR‐223‐3p/FBXW7 signaling pathway affects neovascularization in diabetic retinopathy. Mol Cell Endocrinol. 2019;498:e110541. 10.1016/j.mce.2019.110541 31415795

[38] Huang C , Li Z , Li N , et al. Interleukin 35 expression correlates with microvessel density in pancreatic ductal adenocarcinoma, recruits monocytes, and promotes growth and angiogenesis of xenograft tumors in mice. Gastroenterology. 2018;154(3):675‐688. 28989066 10.1053/j.gastro.2017.09.039

[39] Patel VG , Oh WK , Galsky MD . Treatment of muscle‐invasive and advanced bladder cancer in 2020. CA Cancer J Clin. 2020;70(5):404‐423. 32767764 10.3322/caac.21631

[40] Sonpavde G , Bellmunt J . Bladder cancer: angiogenesis as a therapeutic target in urothelial carcinoma. Nat Rev Urol. 2016;13(6):306‐307. 27071455 10.1038/nrurol.2016.69

[41] Baeriswyl V , Christofori G . The angiogenic switch in carcinogenesis. Semin Cancer Biol. 2009;19(5):329‐337. 19482086 10.1016/j.semcancer.2009.05.003

[42] Yuan A , Chen JJ , Yao PL , Yang PC . The role of interleukin‐8 in cancer cells and microenvironment interaction. Front Biosci. 2005;10:853‐865. 15569594 10.2741/1579

[43] Mian BM , Dinney CP , Bermejo CE , et al. Fully human anti‐interleukin 8 antibody inhibits tumor growth in orthotopic bladder cancer xenografts via down‐regulation of matrix metalloproteases and nuclear factor‐kappaB. Clin Cancer Res. 2003;9(8):3167‐3175. 12912969

[44] Deissler H , Deissler H , Lang S , Lang GE . VEGF‐induced effects on proliferation, migration and tight junctions are restored by ranibizumab (Lucentis) in microvascular retinal endothelial cells. Br J Ophthalmol. 2008;92(6):839‐843. 18511543 10.1136/bjo.2007.135640

[45] Jia Y , Qin T , Zhang X , et al. Effect of bevacizumab on the tight junction proteins of vascular endothelial cells. Am J Transl Res. 2019;11(9):5546‐5559. 31632528 PMC6789270

[46] Huang W , Xing Y , Zhu L , Zhuo J , Cai M . Sorafenib derivatives‐functionalized gold nanoparticles confer protection against tumor angiogenesis and proliferation via suppression of EGFR and VEGFR‐2. Exp Cell Res. 2021;406(1):e112633. 10.1016/j.yexcr.2021.112633 34089726

[47] Khanjani S , Terzidou V , Johnson MR , Bennett PR . NFkappaB and AP‐1 drive human myometrial IL8 expression. Mediators Inflamm. 2012;2012:e504952. 10.1155/2012/504952 PMC3364596 22685373

[48] Robinson KF , Narasipura SD , Wallace J , Ritz EM , Al‐Harthi L . Negative regulation of IL‐8 in human astrocytes depends on beta‐catenin while positive regulation is mediated by TCFs/LEF/ATF2 interaction. Cytokine. 2020;136:e155252. 10.1016/j.cyto.2020.155252 PMC7554258 32818703

[49] Dong Y , Li X , Yu Y , Lv F , Chen Y . JAK/STAT signaling is involved in IL‐35‐induced inhibition of hepatitis B virus antigen‐specific cytotoxic T cell exhaustion in chronic hepatitis B. Life Sci. 2020;252:e117663. 10.1016/j.lfs.2020.117663 32302624

[50] Yang C , Mai H , Peng J , Zhou B , Hou J , Jiang D . STAT4: an immunoregulator contributing to diverse human diseases. Int J Biol Sci. 2020;16(9):1575‐1585. 32226303 10.7150/ijbs.41852 PMC7097918

