# Supplementary material for: Occludin facilitates tumour angiogenesis in bladder cancer by regulating IL8/STAT3 through STAT4
Source: J Cell Mol Med. 2022 Feb 27;26(8):2363–76. doi: 10.1111/jcmm.17257 (PMC8995457; doi:10.1111/jcmm.17257)
Supplement: Supplementary file 1 — Fig S1 [file JCMM-26-2363-s001.docx]

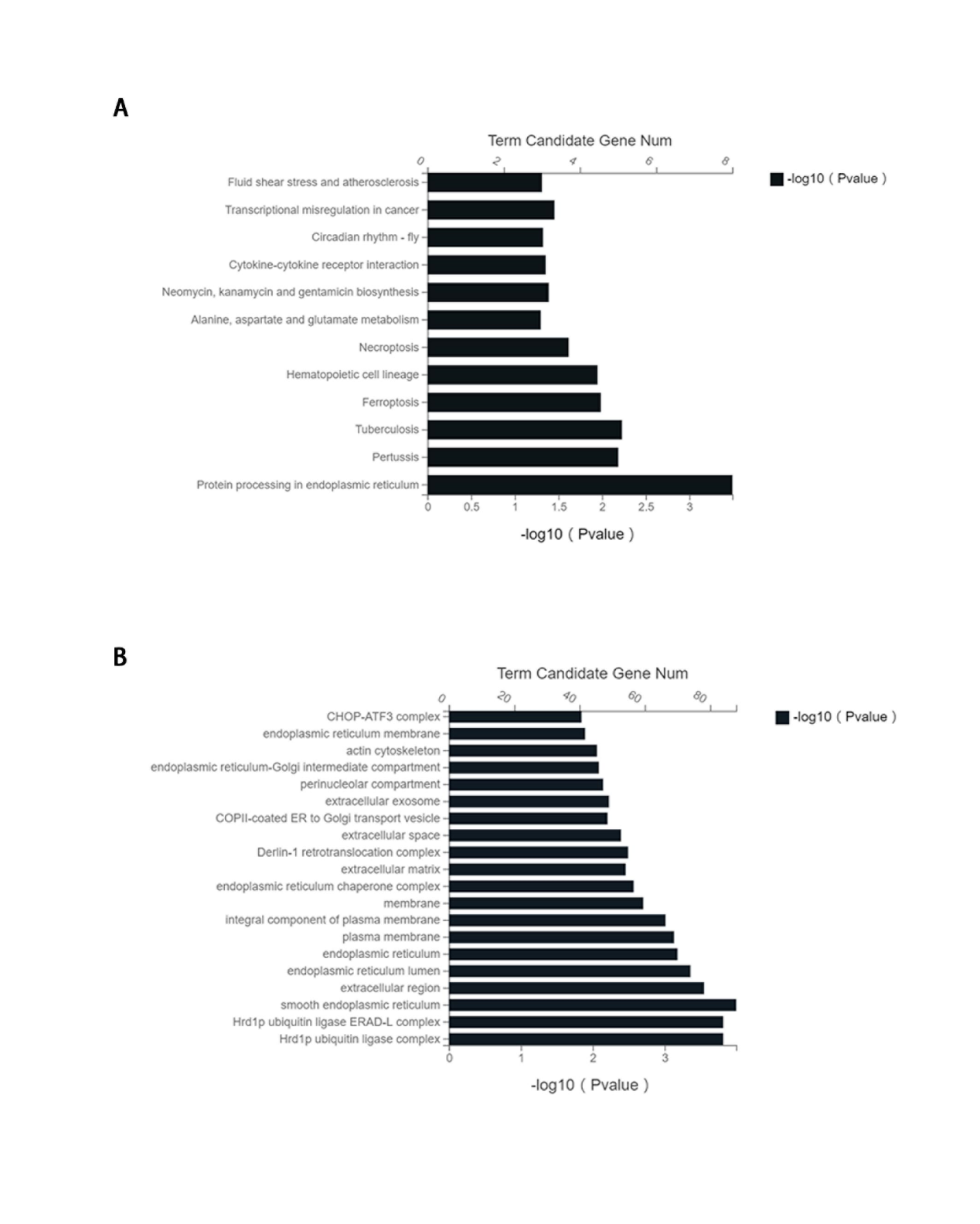


**Supplemental Figure 1** 190 overlaps of the enrichment of the DEG path by means of the enrichment of the KEGG pathway. A, Overlaid DEG function based on FDR value a bar graph showing the set of genesmolecular pathways. B, A bar of extracellular gene sets. Results are showed as the mean±SD. *0.01< *p* < 0.05; **0.001< *p* < 0.01; ****p* < 0.001
